# A New Replicator: A theoretical framework for analysing replication

**DOI:** 10.1186/1741-7007-8-21

**Published:** 2010-03-10

**Authors:** István Zachar, Eörs Szathmáry

**Affiliations:** 1 MTA-ELTE Theoretical Biology and Ecology Research Group, Pázmány P sétány 1/C, H-1117 Budapest, Hungary; 2 COLBUD Collegium Budapest, Szentháromság u 2, H-1014 Budapest, Hungary; 3 The Parmenides Foundation, Kirchplatz 1, D-82049 Munich/Pullach, Germany

## Abstract

**Background:**

Replicators are the crucial entities in evolution. The notion of a replicator, however, is far less exact than the weight of its importance. Without identifying and classifying multiplying entities exactly, their dynamics cannot be determined appropriately. Therefore, it is importance to decide the nature and characteristics of any multiplying entity, in a detailed and formal way.

**Results:**

Replication is basically an autocatalytic process which enables us to rest on the notions of formal chemistry. This statement has major implications. Simple autocatalytic cycle intermediates are considered as non-informational replicators. A consequence of which is that any autocatalytically multiplying entity is a replicator, be it simple or overly complex (even nests). A stricter definition refers to entities which can inherit acquired changes (informational replicators). Simple autocatalytic molecules (and nests) are excluded from this group. However, in turn, any entity possessing copiable information is to be named a replicator, even multicellular organisms. In order to deal with the situation, an abstract, formal framework is presented, which allows the proper identification of various types of replicators. This sheds light on the old problem of the units and levels of selection and evolution. A hierarchical classification for the partition of the replicator-continuum is provided where specific replicators are nested within more general ones. The classification should be able to be successfully applied to known replicators and also to future candidates.

**Conclusion:**

This paper redefines the concept of the replicator from a bottom-up theoretical approach. The formal definition and the abstract models presented can distinguish between among all possible replicator types, based on their quantity of variable and heritable information. This allows for the exact identification of various replicator types and their underlying dynamics. The most important claim is that replication, in general, is basically autocatalysis, with a specific defined environment and selective force. A replicator is not valid unless its working environment, and the selective force to which it is subject, is specified.

## Background

An extensive reformation of the replicator definition is needed. There are two main reasons for proposing a more exact definition. First, current definitions and classifications (see, for example, [1-17]) cannot discriminate effectively between gene-like entities (being sufficiently abstract) and organisms, or gene-like entities and *s*imple autocatalytic cycle intermediates (SACIs; for example, glycolaldehyde in the formose reaction [18]). There is obviously some difference between these entities concerning their copying dynamics and exhibited similarity between parent and offspring entities. However, present definitions usually use indefinite terms such as 'almost identical' [3], 'largely intact structure' [9], 'relevant aspects' [2,5], 'relevantly similar' [15] and 'same kind' [11] (see Griesemer's comment [[6], p. 73], about vagueness and the validity of 'same kind' [[19], p. S359]). These definitions do not specify the difference exactly, nor do they clearly mark the borders of the entity-space that could be labelled as replicators. Secondly, some current definitions have been formulated to include only the genes but not the memes (for example [8,9]) or other putative replicators of culture. Memes (the gene-analogue cultural replicators introduced by Dawkins [3,4]), as we refer to them, are cultural traits, that are replicated by copying/imitation (for example, words, concepts, songs, and so on) [20-22].

Relevant aspects surely count in replication. However, exactly which features are relevant? To what extent should these features be similar to each other? How can these features and amounts be qualified or quantified? In which case are two replicators identical? What changes cause the loss of identity of offspring compared to parents? Since replication is about multiplying an entity in such a way that the resulting entities are similar to parent entities, this question is of utmost importance and the loose terms cited above cannot show the exact nature of the identity.

DNA replication (as the standard replicative system is usually referred to) has specific aspects which may be used as a basis for the definition of replicators: two entities are made using only one and, ideally, they are identical. If the DNA is located in a germline cell, it is potentially the originator of an indefinitely long line of descendants (see germline replicator [4]). However several aspects of DNA replication do not apply to every (real or putative) replicator. From a strictly chemical point of view Orgel has pointed out that 'few of the features of RNA replication are essential for a general replication model' [[23], p. 204]. Material overlap (understood as a generative method where some physical part of the offspring entity was part of the parental entity earlier), for example, is clearly not a universal necessity of replication. Anything could mediate the information-transmission from parent to offspring; it need not be the parent itself. Cultural traits, if they are considered replicators, would exclude material overlap (see [6]) from the universal features of replication: in case of memes there is undoubtedly no overlap of physical entities.

Nevertheless, specific differences among replicative systems do cause changes in the dynamics of replication. It follows that a universal formalism should focus on the general aspects that do not change our basic concept of replication. Therefore, it is important to formulate the new definition in a general and formal way that clearly describes what is common and what is different among presupposed replicators, and which can include or exclude candidates accordingly. Presupposed replicators are genes, memes, ribozymes and several other candidates such as autocatalytic protein nets [24], prions [25], membranes [26-28], chromosomes [3], genomes [3], organelles (for example, plastids [11]), organisms, kin (Dawkins [29] describes the individual, obviously the genome, as a genetic octopus extending to relatives), an so on. [Note that well-discussed candidates (DNA, genes, chromosomes, and so on) are not referenced here due to the large number of publications.]

Earlier definitions are either too narrow (for example [8,9]) or too broad (for example, definitions based on Muller's multiplication-heredity-variability criteria [12] or, later, by Maynard Smith [10,30]). The former, by sticking to structure, do not allow the inclusion of memes, while the latter allow the inclusion of, for example, organisms or even supra-individual entities. The general intuitive requirements that we suggest must be met by replicators (based on present knowledge, existing definitions and personal views), are:

1. Potentially autocatalytic mode of generation.

2. Potentially above-chance similarity between parent and offspring.

3. Ability to pass on some information to offspring.

4. The definition has to include genes and memes, and possibly other entities as well (as it was Dawkins [3] who has introduced the term replicator, it seems reasonable to focus on these two entities he has suggested).

5. The definition must be able to distinguish between multiplying entities: genes, memes, simple chemical cycle intermediates, organisms and higher level entities (for example, kin groups) in a clear and exact way.

The general aspects that have been extracted from most of the present definitions should be considered as the general, vague concept of a replicator. Those components of present definitions that are proved (or will be proved) not to be necessary (like material overlap), should be left out. Thus, four questions are raised that the new definition should be able to answer clearly:

1. What is the difference between entities such as genes, organisms, SACIs and other reproducing entities?

2. What are copying, heredity and modularity. What part do they play in case of replicators?

3. What is similarity? When can we say that two replicators are identical?

4. What are the common and differing aspects of genes and memes? What is the definition that puts memes and genes into the same box but organisms into a surrounding larger one (this setup of entities follows Dawkins' definition [3,4])?

## Results and discussion

### Multiplication

**Figure 1:**
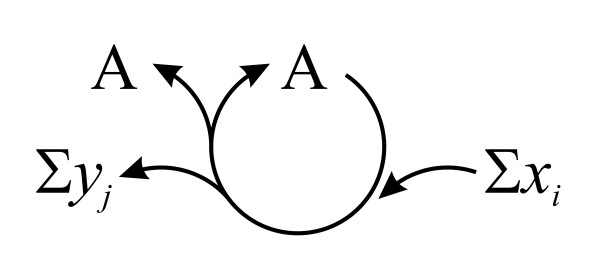
**An autocatalytic cycle of the form Σ*x*_*i *_+ A —①→ Σ*y*_*j *_+ 2A (following Gánti's notation **[68]). *x*_*i *_and *y*_*j *_denote all the necessary input and waste molecules respectively, of one turn of the cycle.

The first criterion of a replicator is unquestionably multiplication. However, what exactly is multiplication? In what aspect is it more than the mere increment of the number of instances? How does it relate to autocatalysis and similarity? Consider a simple case where a glass of water is divided into two halves. No one would consider it to be multiplication. However, there are two portions of water where there was only one before and the two bear almost complete resemblance (with minor statistical fluctuations) to the original (except in volume). The difference between dividing a glass of water and a true autocatalytic cycle lies in the stoichiometry. Without input, a litre of water divided would not give two litres. The 'reaction' cannot be rendered to be cyclic, nor autocatalysis. Thus, it is necessary that some material is fed into a presupposed autocatalytic cycle (Figure 1).

**Figure 2:**
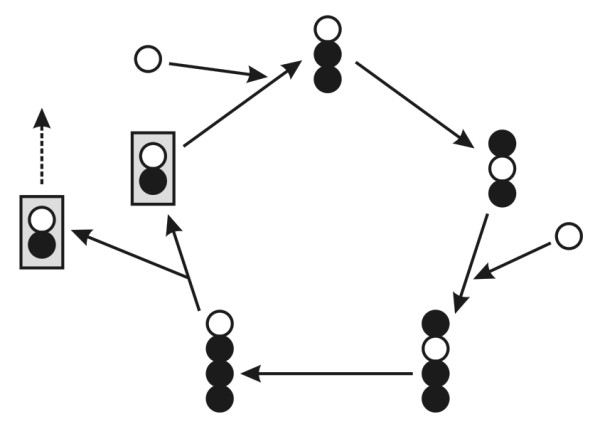
**A truly autocatalytic cycle: the autocatalytic core of the formose cycle **[18]. The framed molecule is glycolaldehyde.

What is characteristic of an autocatalytic entity or cycle (for example the formose reaction of Figure 2[18]) is that ' [it] can arise only if there is a pre-existing structure of the same kind in the vicinity' [11]. This expansion requires some clarification of the *sameness *of the original and new entities. It is obvious that if the new 'A' does not resemble enough the old 'A', then the cycle will not close. However, the two 'A's can be totally different in, for example, structure, as long as they are functioning sufficiently similar to allow the cycle to close. Indeed, a new cell is not exactly the same as the old cell but both have the potential to produce more cells. It is tempting to say here that the parent and offspring must be similar in some 'relevant aspects', but it just shifts the undefined to a further point that must be reached nevertheless. For the moment, let us just say that the old and new 'A's must be *equivalent *in some way, until this equivalence is defined exactly in the following paragraphs.

Pre-existence is also important from the viewpoint of causality. Hodgson and Knudsen [7] stated concerning photocopies that 'the original is causally implicated in the production of the copy, in the weak sense that without the original the copy could not exist'. This simply means that any autocatalytic cycle automatically fulfils causality - that is, that there is no need to explicitly specify it as a criterion of replication.

To conclude, dividing a glass of water is not an autocatalytic process and, thus, multiplication (based on autocatalysis) requires three specifications: (1) there must be some input of matter (for example, molecules used as building blocks), which is used to create the new entities; (2) parent and offspring must be equivalent, that is they must be based on the function of the entity in the cycle; (3) one turn of a cycle should produce more offspring than the number of parents (for example, a regenerated parent and a surplus offspring). Autocatalysis and the equivalence of parent and offspring entities are closely linked and one cannot be present without the other. If none of the products of a reaction is functionally identical to the original, then there is no cycle at all.

In order to capture the essence of equivalence, and to decide about whether some structural, functional or informational feature should be the distinctive factor among entities, we must ask the following question: During the lifetime of an entity for whom or what is it relevant to distinguish between non-identical entities? The answer is obviously some selective force. The identification of entities should be based on selection. Without selecting anything among entities, there is no way to search for the differences among them.

Therefore the following definitions are given:

1.1 *Selection *is a process, acting on a particular population of entities in a particular environment that sorts entities according to a function of the entities. It can be thought of as a single sorting event, during which some entities cease functioning while others persist and can be iterated indefinitely. This is based on the definitions of selection by Hull [[9], p. 408] and of sorting by Vrba [[31], p. 117].

1.2 *Equivalence*: Two entities are equivalent under a particular selective force in a particular environment, if this selection process cannot sort among them better than random. The equivalence of two entities is denoted as *e*_1_~*e*_2_.

1.3 *Phenotype *is the function of entities selection discriminates about. The phenotype defines the level of selection as well.

The concepts of *selection for *(a property) and *selection of *(objects) given by Sober [[32], p. 99] indicate the same setup: selection is a general process that can act without multiplication.

What exactly is the 'relevant aspect' of sameness is quite irrelevant, until the parent and offspring are equivalent under a certain selective force. This is what Sterelny *et al*. [[15], p. 396] tried to capture when they referred to copying as a teleological notion in their definition of replication. To see why this definition of equivalence is effective, let us imagine a population of autocatalytic cycles. The product of the cycle changes to a new entity in order to introduce a new cycle. The original and new cycles can be different, but any difference will only make sense if this difference is expressed at the level of selection. If it is not, then selection will not cause differential survival. Either naturally or artificially, there is a selective force which explicitly discriminates between cycles. Even more striking is the example of triplet-groups: UCA and AGU are completely different codons, although they both code for serine as they are phenotypically identical. Of course, this neutrality is only valid in case of a single or a few selective events, but not necessarily in a larger timeframe where iterated selection might induce evolution (non-trivial neutrality will be discussed later).

These terms can be expressed in a more formal way using equivalence relations (see, for example, [33]). Selection partitions the set of all entities *E *into equivalence classes. Entities of the same class are identical from the viewpoint of selection. Thus, selection defines an equivalence relation *S *(or ~) on *E*. The phenotype is that function *p*: *E *→ *p*(*E*) for which it is true, that ∀ *e*_1_, *e*_2 _∈ *E*: *e*_1 _*S e*_2 _⇔ *p*(*e*_1_) = *p*(*e*_2_). The phenotype function thus maps entities to such a set that, if partitioned by the equality relation, has the exact same structure as the partition of *E *implied by the relation *S*.

In order to deal with the actual multiplication of an entity (growth in number), let us start from a general stoichiometric notation:(1)

**Figure 3:**
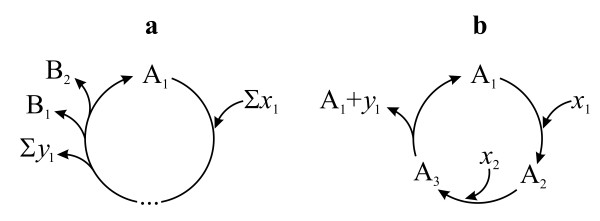
**(a) A simple cycle, that can be autocatalytic if B_1 _~ A and/or B_2 _~ A; or non-autocatalytic if B_1 _!~ A !~ B_2_, where ~ is the equivalency relation**. (b) A valid autocatalytic cycle.

In an autocatalytic system (Figure 3) *n *> 1 elements of the set of products {B_1_, B_2_, ..., B_*k*_} are equivalent to A (where no *x*_*i *_and *y*_*j *_is equivalent to A or any B_*k*_). If *n *= 0 then no B_*k *_is equivalent to A. The reaction cannot be iterated, since all A-s are used up (that is, it is not a cycle) and A is not multiplied. Iff *n *≥ 1, the reaction can close to a cycle. With *n *= 1 there is no multiplication, but sequential replacement where the overall concentration of A is constant in time. It can, however, be depleted easily due to potential side reactions. Nonetheless, the system is capable of processing A any time that it is fed with Σ*x*_*i *_*and *an existing A is already present. Simple non-autocatalytic cycle intermediates (SNCIs) belong to this class. Finally, if *n *≥ 2, then A is an autocatalytic entity (*n *giving the order of the autocatalytic system).

From a dynamical viewpoint the growth rate *g *of reproducing entities must be larger than the spontaneous dissociation rate *d *in order to overcome sequential replacement or depletion (see [[11], p. 33], [34]), otherwise entities would decay with equal or faster rate than they multiply. It is a potential criterion for replicators to be in a stable state long enough to be multiplied at least once (*g *>*d*, see Dawkins' longevity criterion [3]). Although this kinetic requirement is implicitly present in the notion of autocatalysis, if the cycle cannot produce a surplus of A, because the decay ratio (either due to side reactions or due to the reverse reaction of production) is faster than the ratio of production, the process is effectively not autocatalytic.

From this viewpoint, multiplying entities with *d *= 0 and *g *> 1 would be immortal in the sense that a *population *of such replicators would not be depleted at all. A lack of decomposition (mortality) does not exclude selection or even evolution, as Lewontin [[35], p. 1] puts it: 'Natural selection occurs even when two bacterial strains are growing logarithmically in an excess of nutrient broth if they have different division times' - that is, bacteria are practically immortal in the timeframe of the experiment but selection still happens. Szathmáry and Maynard Smith [36] showed that the relative concentrations in the case of unlimited resources change just as in the case of a limiting environment, resulting in the extreme dilution of the replicator with the smaller replication rate in the limit.

To summarize: multiplication, as a prerequisite for replication, is simply the phenomenological expression of autocatalysis:

1.4 *Multiplication *is an autocatalytic process, where some of the products of the process are equivalent to the original entity - that is, functional identity is preserved (stoichiometrically *n *> 1, kinetically *g *>*d*).

The criteria for multiplication are as follows: the entity should be stable long enough to reproduce at least once and the outcome of the process should contain more than one parent-equivalent entities. The similarity necessary for the system to run successively was also defined as an equivalence relation of a parent and an offspring entity under a selective process. Multiplication defined here equals autocatalysis, since there is equivalency only where the cycles are autocatalytic. Note that (according to Lewontin [35]) if autocatalytically growing entities exhibit differences in their phenotypes, they are subject to selection - that is, they are *units of selection *(this will be discussed in detail under the section on Evolution).

### Variability

With the equivalence of phenotypes we indirectly introduced variability. Two entities may vary in structure significantly and still be equivalent (due to phenotypes) until selection cannot distinguish them. Hull ([[37], p. 32], following Williams [38]) has pointed out the same relation, although he did not go further than genes: 'two genes are similar enough to count as the same replicator if they react similarly to similar selection pressures'. Again, selective pressure here is understood as it was defined earlier (point 1.1): as a single sorting event. Two genes which are identical from the viewpoint of an immediate sorting event can still have different evolutionary potentials.

The problem of variability, similarity and 'relevant aspects'" was discussed by Aunger [2] (the Queen and British postage stamps example). However, his solution of narrowing the causality clause (and introducing the notion of lineage as a causal linkage) is not satisfactory. Causally linked parent and offspring entities, like original and replica DNA sequences may still have the same base-order, although they may be differently methylated. From the viewpoint of the protein they code for they are the same, while from the viewpoint of the gene-regulation they are completely different. Even if causality is present, the similarity of parent and offspring is not obvious. Sterelny *et al*. [[15], p. 396] concluded in their replicator definition that 'A fossil of a leaf is not a copy of a leaf' - although there is a causal link. One can say then, that the relevant aspect is the one that is common in parent and offspring - that is, the base order of the DNA. However, this is exactly the part which tends to change during evolution. In Aunger's Queen and stamps example, the relevant aspect is nothing more than the lateral silhouette of the head of the Queen reprinted in each British stamp. The problem with the Queen's head is not a problem of reproduction: the lateral silhouette does get reproduced from time to time. The difference is the lack of autocatalysis: the depicted head will not induce new cycles producing new heads (and that is why there is no causal link between parent and offspring here).

So if the equivalence relation (defined by selection) is used to decide identity, then what are those changes which shift an entity in the phenotype space to yield a different phenotype? A change that ruins the whole cycle obviously affects the phenotype: the offspring will not be able to function as did the original cycle. However, what if the product of the cycle changes and the cycle can go on? Since heritability has not yet been introduced, let us assume that the change is a one-time fluctuation. Such fluctuations are lost in the next turn and the cycle goes on with the original intermediates.

What happens if the product changes and, due to inheritance, so does the cycle?: a new entity induces a new cycle that can produce the new entity rather than the original. The new and old entities are equivalent if, and only if, their phenotypes are equivalent. Thus, if their replication rates are the same, from the viewpoint of selection, they will be equivalent. What happens if they have the same growth rate, but the old one was an ineffective chemical drug while the new one cures a disease? The selective force in this case is obviously not based on growth rate but on their effect on the given disease and on the grant of the laboratory that has synthesized it.

Alternatives of a cycle can be 'heritable' as well, even if no direct template-replication is involved. Consider, for example, the (hypothetical) alternative reductive citric acid cycle, where some standard components are substituted by thioanalogues [39]. In this case, the use of the thioanalogue instead of citrate in a specific reaction step is not due to template-copying (matching) but due to the fluctuations in external factors, such as the availability of resources, nature of the environment, temperature and so. Individual intermediates are replicating exactly, without the possibility to inherit changes (non-informational holistic [25] or processive [16] replicators). The cycle as a whole, however, may exhibit attractor-based heredity [40,25], if the system can settle mainly using the analogues. Holistic replicators in such systems may, therefore, convey one bit of information (presence or absence).

### Heredity model

In order to deal with real heritable changes, inheritance must be introduced. Let us start from a simple distinction of replicators. Szathmáry and Maynard Smith [[17], p. 201] have introduced the terms 'unlimited' and 'limited' heredity as attributes of replicators: 'Limited hereditary replicators, owing to their structural peculiarities, can exist and be replicated in only a few stable states, whereas unlimited hereditary replicators can encode for a practically infinite set of varieties'. Later Szathmáry [[41], p. 2] pointed out an important property of this distinction: 'Limited heredity is context-dependent, since we require that the number of possible types should be smaller than the number of actual individuals present'. Thus, the unlimited/limited attribute is a binary distinction of replicators, depending on two factors of the actual context: the population size (external) and the quantity of variability that can arise in any one entity (internal).

**Figure 4:**
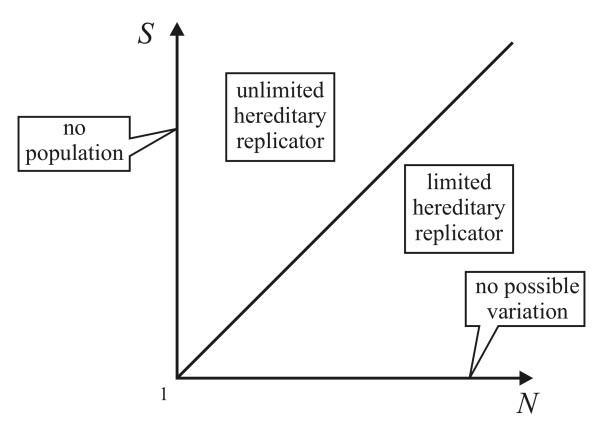
**Domains of the *S N *space**. *S *is the number of possible states of a replicator, *N *is the number of individuals present in a population of such replicators.

Let *S *be the number of possible different, stable states of an entity and *N *the number of actual individuals (size of population). The model, therefore, thus depends on these two parameters which (without any assumptions) can have integer values from one to infinity. Entities can be classified as replicators with limited or unlimited hereditary potential according to *S *and *N *(Figure 4).

At *S *= 1 there is no variation and *N *= 1 means there is no population. With *N *>*S *the population is larger than the number of possible variants - replicators have a limited hereditary potential. However, if *N *≤ *S *the population is smaller than the possible number of variants - replicators have unlimited hereditary potential. Since both variables are quantifiable it seems rational to include extremes in the investigation. Following this, *S *= 1 (especially *N *= *S *= 1) can be considered as an extreme class of replicators, exhibiting exactness - that is, there is no evolutionary potential at all. Note that, at *S *= 1, there is no stable state of the entity beside its present state - that is, there can be no variation in this entity. This clearly fits SACIs. In order to prove it, one must assume that an appreciably long DNA chain is an unlimited hereditary replicator, as the population size is smaller than the possible number of types. If we decrease the chain-length below one point (where the number of possible types equals the size of the population), the oligonucleotide becomes a limited hereditary replicator. If the length is further shortened, ultimately we would end up with a single nucleotide, which would be a SACI (although single nucleotides cannot replicate, due to the weakness of the hydrogen bonds). Thus, it can be stated that if we allow the unlimited/limited distinction among replicators, and think of those of extremely limited (or even no) hereditary potential as replicators, we would also have to include SACIs in the group of replicators. This is why Szathmáry [16] has introduced the term '*holistic *(*processive*) *replicator' *to denote primarily non-modular autocatalytic cycle intermediates.

The inclusion of SACIs would imply that the term *replicator *is not the same as *unit of evolution *(*sensu *Muller [12] or Maynard Smith [10]), since exact replicators are not subjects to evolution (no variability or heredity - that is, are not genetic systems *sensu *Orgel [23]). In our opinion, any multiplying entity which fits the multiplication criteria (1.4), but lacks evolvability, could still be considered a replicator: this also agrees with the vast (and growing) experimental chemical literature. Of course, if the cycle consists of multiple exact replicators, there still can be heritable information (for example, presence/absence of specific intermediates). However, that is not a property of the intermediates, but a property of the cycle as a whole (like the distribution of intermediates).

There are three reasons for this. First, Szathmáry and Maynard Smith [17] have highlighted the fact that there are limited, or even non-hereditary replicators with limited or no evolutionary potential at all, which are part of the same continuum. Secondly, the ideal gene would be an exactly replicating entity if proofreading was faultless (this surely happens quite often), prohibiting short-term microevolution. The third reason follows on from this fact. A diverse population of holistic replicators lacking evolutionary potential still can be the subject of selection. If there is competition, and difference in replication rates, one competitor will possibly wipe out the other, even if there is no microevolution occurring. Thus, selection can act on a population of non-evolutionary replicators.

Exact replicators are those which cannot pass on any information (acquired changes) to their offspring. These entities are exactly those described by Orgel [23] as 'non-informational replicators' (in contrast to informational ones), which are usually non-modular and are not able to hold (biologically important) information. Furthermore, since no information can be changed and inherited by them (as stated by Orgel) they cannot evolve by natural selection. In contrast, those entities that do possess some evolutionary potential are limited or unlimited hereditary replicators with limited or open-ended potential for evolution. This suggests that evolvable replicators are a subgroup of replicators. Table 1 summarizes the distinction of replicators based on the hereditary potential.

**Table 1:** Advanced classification of replicators according to hereditary potential

		**Evolution**	**Variability**	**Heritable variability**	**Modularity**	**Heritable information**	**Examples**
	
*Modular*	Unlimited hereditary replicators	Units of open-ended evolution	High	Primarily template-based	Polimodular	Combinatorial	Replicators with sufficiently large number and variability of modules
	Limited hereditary replicators	Units of limited evolution	Low		Oligomodular		Modular SACIs
	
*Holistic*	Exact replicators	Can be units of limited evolution, as members of a system	No	Only attractor-based	Non-modular	1 bit	Replicators which cannot be replaced
	Variable replicators		Some		Modular	(presence/absence)	Replicators which cannot pass on changes

SACI = sample autocatalytic cycle intermediate

### Nests are non-informational replicators

The very effective distinction of limited and unlimited hereditary replicators [17] implies that SACIs are extremely limited-hereditary-replicators: with no heredity at all (or only attractor-based). This is in close relation with what Orgel [[23], p. 203] suggested: 'All replicating systems are, by definition, autocatalytic and all autocatalytic systems result, in some sense, in replication'. If we compare SACIs with genes (as replicators), we can extract their common and distinctive features: (1) they both have a similarity between parent and offspring; (2) both are autocatalytic; (3) SACIs do not have any heritable parts; and (4) similarity in SACIs is not because of copying (there is no information transfer from parent to offspring). The process of copying (matching) is exactly defined under the Heredity section.

Of course, the major difference is in the amount of information SACIs and genes can maintain or transmit. This incorporates the fact that genes do have a heritable part (which, based on evolution, allows a combinatorial search space), while SACIs usually do not have any information that can be passed on. Of course, even simple molecules may provide information as part of a larger system. The absence or presence of a particular autocatalytic cycle in a cell obviously has serious consequences. Furthermore, any novel autocatalytic cycle may propagate to offspring cells, making the change therefore hereditary. This by no means indicates that any change applied to a SACI (the intermediate of any such cycle) is inherited by its offspring molecules. SACIs, therefore, are non-informational. According to point (4), Dawkins' copying criteria should be dropped in order that SACIs can be included. Allowing all SACIs to be replicators implies that autocatalytic entities with no heritable part can also be replicators.

The irritating problem here is that this statement completely fits with, for example, bird nests/eyes/organisms (see [15,42-44]), if we consider such things to be parts of autocatalytic cycles. This is basically true, because the earlier presence of nests/eyes/organisms catalyze the reappearance of similar entities in the following generations. Also one cannot deny the fact that, as birds populated Earth, so did nests, in ever-increasing numbers, although they were involved less directly. In one word, nests do fit the multiplication criterion (1.4).

It seems, then, extremely alluring to exclude SACIs from replicators, in order to also get rid of unwanted nests. However, if we follow this line of thinking, we would have to exclude extremes (non-informational replication) from the limited/unlimited hereditary model. We would also lose the possibility of using the same definition for perfectly replicating real evolutionary replicators, or the chemical predecessors of them, with limited or no heredity at all. This seems an unnecessary cut in replicators. On the other hand, we would have to allow, for example, nests to be replicators, which is a strongly debated claim (see [15,42-44]). This is a circular problem that obviously cannot be solved at this point.

Maynard Smith and Szathmáry [[11], p. 20] state that: 'it is important to emphasize that autocatalysis is not the same as replication. For replication, it is not sufficient that an *A *gives rise to two *A*s: it is also necessary that, if the *A *is replaced by *B *(or a *C *or a *D*), then the cycle should give rise to two *B*s (or *C*s, or *D*s). In autocatalysis, there is no variation, and hence no heredity. Autocatalysis is an important first step towards replication, but it is not the whole road'. At this point they defined replication in terms of heredity and variability and any entity lacking these should be excluded from replicators. This statement therefore would exclude not only nests, but SACIs (and non-informational replicators) as well from replicators. However, it is not contrary to their previous statement (SACIs are processive replicators [17]): it is just a slight specification of a term. It is, somehow, a matter of arbitrariness whether or not one considers non-informational replicators to be real replicators. Nonetheless, the problem of the similarity between SACIs and nests, and the emerged situation of whether either both or neither of them should be a replicator, is still valid. In order to evaluate this problem, let us turn to autocatalysis again.

### Autocatalysis in depth

In order to investigate the problem of nests, it is necessary to look at the structure of autocatalytic molecular networks, since they are the most primitive naturally autocatalytic systems, and the lessons drawn will turn out to be fruitful in the subsequent analysis of other systems in this paper.

The autocatalytic core of the formose cycle [18] (Figure 2) is the simplest case and it has the special feature that it works without enzymatic aid. It is analogous to the reproduction of bacteria or the fission of yeast. An important lesson is that the different molecules in the cycle can be seen as analogous to phases of the lifecycle of a reproducing cell: this is the reason why the cycle can be ignited alternatively by any of these intermediates, just as it is by pure convention that we list the phases of the typical eukaryotic cell's cycle in the order *G*_1_, *S*, *G*_2_, *M*.

**Figure 5:**
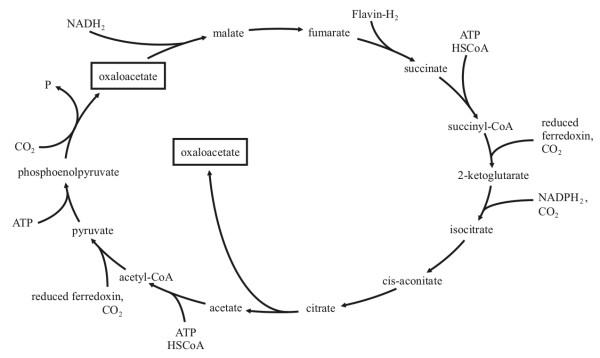
**The reductive citric acid cycle**. Note the asymmetric branches leading to the two molecules of oxaloacetate.

Not all such autocatalytic cycles are as simple as this, however. The (enzymatically catalyzed) reductive citric acid cycle is asymmetric: whereas after one turn all intermediates are doubled and one oxaloacetate is produced differently from the other (Figure 5).

**Figure 6:**
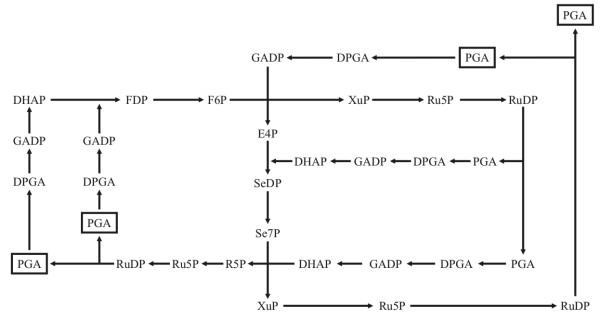
**The Calvin cycle**. It is clear that it is autocatalytic, but it is also clear that one molecule of 3-phosphoglycerate is not enough to ignite the system. The minimum number of molecules is two (for example, one molecule of Se7P and one DHAP), provided that all the obligate enzymes are present. (Se7p = sedulose-7-phosphate; DHAP = dihydroxi-acetone-phosphate). From Szathmáry [16].

The Calvin cycle is also autocatalytic [45] but it is a complex network of sugar phosphate molecules. The most important feature for us here is that at no stage can it be reduced to one intermediate that could create the whole network: there should be at least two or three molecules present so that all the reactions can commence (Figure 6). Thus, it can be somewhat misleading to say that, for example, 3-phosphoglycerate is a replicator: in fact, three molecules of 3-phosphoglycerate form sufficient autocatalytic seed [46]. Therefore, the Calvin cycle, and the formation of PGA, is autocatalytic but the replication ratio of PGA is 4/3 instead of two as in the case of the formose cycle regarding glycolaldehyde.

Gánti [47] postulated that the metabolism of present-day living systems rests on an autocatalytic set of molecules that one cannot omit from the system, since all the genes and enzymes fail to contain the relevant chemical information. This postulate has been proven for all organisms where sufficiently detailed knowledge of metabolism is available [46].

Here a remark is in order. Although, by themselves, such simple molecular replicators are non-informational (similar to a gene with only one allele and perfect accuracy), in the context of a larger system they carry information that, as in the case above, cannot be provided by the rest of the system. In fact, the obligate metabolic autocatalysts are part of the 'genome' in the wider sense. Nevertheless, no change applied to such a molecular replicator is inherited in the successive turns of the metabolic cycle, but the presence or absence of such cycles may be inherited in the successive cycles of the cell.

**Figure 7:**
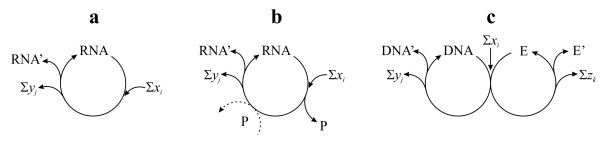
**Autocatalytic nucleic acid systems**. (a) RNA as a true replicator. (b) proteins (P) appear as side-products of RNA replication, which facultatively catalyze the formation of the new RNA molecule (dashed arrow). (c) The present (simplified) relationship between genes (DNA) and enzymes (E), which are both obligatory for the other to be autocatalytic. *x*_*i *_denotes all input materials while *y*_*j *_and *z*_*k *_denote waste materials.

It is instructive to consider autocatalysis of the nucleic acid-protein system from the point of view of structure and evolution. If we assume that there was an RNA world (as many do), then RNA was a true replicator (Figure 7a) and, then, some peptides produced by RNAs appeared that then facultatively aided the replication process (Figure 7b). By now the relationship between genes and enzymes is completely obligatory: none of them can be reproduced without the other (Figure 7c). Hence the only correct statement is that nucleic acids and proteins *together *self-replicate (each is catalytically linked to the other). This does not deny the fact that most of the informational part of the replication rests with the nucleic acids.

The foregoing analysis of metabolic replicator systems helps to clarify the burning issue of whether organs or nests are replicators. It also sheds light on replication, inheritance and niche construction. The clear answer is that by themselves nests or organs are not replicators. Formal stoichiometry of the autocatalytic processes reveals why.

**Figure 8:**
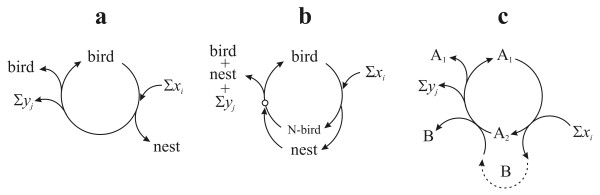
**Autocatalytic system of birds and nests**. (a) Birds as simple catalysts of nests. No nest is needed to produce new birds. (b) The nesting bird phase can only continue if there is a nest present (possibly one that was built by the bird, as it is pictured here). Small circle indicates point where a nest is obligatory. (c) Formal version of (b), with A_1 _as the bird, A_2 _the nesting bird and B the nest. Note: Figure 7(b) is equivalent to (b) and (c) if E is an obligatory catalyst of RNA.

Let us first consider the bird's nest. Suppose that birds can build nests anew. The simplest assumption is that birds act like catalysts (enzymes) in the production of the nests (Figure 8a). This is not strictly true, however, because normally the bird is exhausted and/or is already fertilized, with the eggs growing during the process (for the sake of simplicity we consider only female birds). Thus, we display a nested bird (N-bird) as a different phase in the lifecycle (Figure 8b). It is true that only the N-bird and the nest together can proceed with the 'reactions', the result of which is the production of at least one new bird, which can also build a nest, and so on. The number of birds and nests multiply with time. Looking at the lifecycle one can say that the bird is one phase of a replicator and {N-bird + nest} is another phase. The informational part in this simple example rests with the bird, not the nest. These need not be so: if forms of nest are associated with forms of relevant memes, then there is cultural and technological evolution going on at the same time. In this case, it makes sense to regard the nests as extended phenotypes of genes and memes together.

The case becomes stronger in the hypothetical example when a bird cannot live at all without the nest (of course, this could only be a derived character from a previous evolutionary stage). This would be analogous to the nucleic acid-protein system.

The same logic clarifies the problem whether or not organs are replicators. Since organs form in a special phase of the lifecycle of organisms, it is arbitrary where one places the beginning of the cycle - the zygote is one autocatalytic seed and the organism with all its organs is another one - ultimately it is the whole lifecycle that is reproduced. This may mean that the eye (and the nest) by itself is not a replicator, but stoichiometry makes the situation clear again, as follows.

The net equation of Figure 8c is:(2)

The equation indicates that A_1 _is an autocatalytic entity and B (as a product) is also increased in time. Thus, a possible autocatalytic seed of the cycle is A_1_. (Note that, strictly speaking, one ought to apply cyclic stoichiometry to all these cases.)

**Figure 9:**
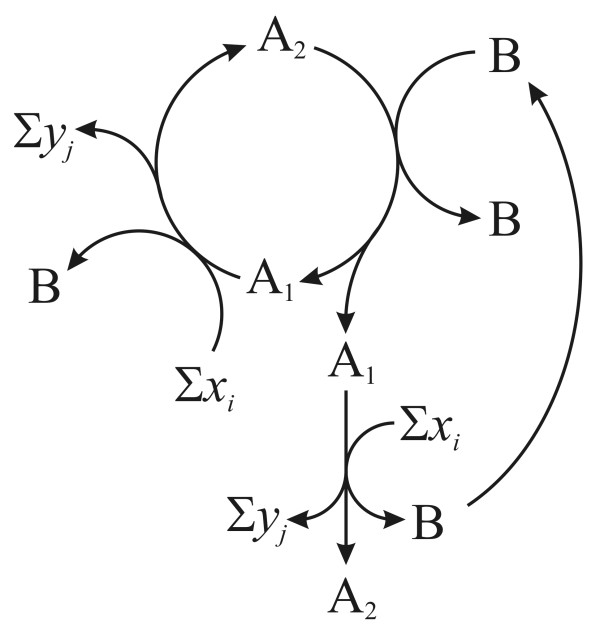
**The same autocatalytic cycle of Figure 8c from the viewpoint of A_2_**.

Gánti [47] has pointed out that, if such an autocatalytic cycle is given, any of the intermediates (A_1_, A_2_, ... A_n_) is doubled during one turn, thus both the cycle and all intermediates are also autocatalytic. Therefore, let us turn the cycle of Figure 8c by 180°, to reflect what happens from the viewpoint of A_2 _(Figure 9).

The basic reactions are unchanged, but the net stoichiometric equation, reflecting the autocatalytic nature of A_2_, is a bit different:(3)

This equation indicates that another autocatalytic seed of the same cycle is the {A_2_, B} set.

If Gánti's abovementioned statement is correct, both A_1 _and A_2 _are autocatalytic, although there is one major difference between them: while A_1 _can start the cycle on its own (provided that Σ*x*_*i *_is present in the environment), A_2 _can only initiate the cycle if B is also present. Thus, while A_1 _is a totally autonomous replicator, A_2 _is a dependent one, depending on its obligatory partner B.

Since B is created as a side product of A, it seems that it is not autocatalytic. However, since A is a cycle, it is always true (disregarding from where the cycle is viewed) that an existing B is required as a catalyst in order to give the cycle one full turn, after which a new B is generated from scratch. Thus, an existing B catalyses *indirectly *(that is, the new B is not created by the original B, there are intermediate steps) the formation of a new B. This is completely independent of the fact whether Bs are needed in stoichiometric amounts or only one B is needed for all steps to be catalyzed in all the parallel cycles, since the total amount of B will be increased; thus, the replication ratio of B will be larger than one. Therefore, since the definition of multiplication (and autocatalysis) fits B, it must be autocatalytic as well, although indirectly.

The strange thing, that the autocatalytic property of B is revealed only in equation (3), is due to the fact that equation (2) informs us *only *about A_1_. Equation (3) deals primarily with A_2_, but the lucky fact is that the net equation of the cycle from the viewpoint of B is exactly the same as equation (3). Thus, as equation (3) does not say much about the role of A_1_, the same way equation (2) does not say much about A_2 _or B.

The bottom line is: in cycle A, A_1 _is an *autonomous replicator *(or obligate autocatalyst), while A_2 _is a B-dependent replicator and B is an *A*_2_*-*dependent replicator (or facultative autocatalysts). This means that if A_1 _is the bird, A_2 _is the nesting bird and B is the nest. Therefore, that nests can also be regarded as replicators, although dependent ones only (and still the more important informational part) is ignored in this model. Thus, analysis can reveal the underlying difference between dynamically similarly behaving replicators.

To conclude, it is of utmost importance in order to distinguish between *obligate *and *facultative autocatalysts *of an autocatalytic cycle. The consequence of this distinction is that, while an entity can be reproduced autocatalytically, it may not be enough to start a cycle: phosphoglycerate (PGA) is autocatalytic in the Calvin cycle, but one molecule in itself is not enough to initiate reproduction as it needs at least three molecules of PGA and all its obligatory catalysts. Any component, which can initiate the cycle, forms the autocatalytic seed of the cycle. Thus (according to Figure 8b) birds are independent replicators, but not nests (or organs): a nest cannot produce one new nest because it cannot start a new cycle on its own. However, as far as a nesting bird is considered as a phase of a replicator, nests must be replicators as well: more precisely, the combination of {nesting bird + nest} form the appropriate autocatalytic seed. In other words, the nest in itself is no less a replicator, than DNA without enzymes.

Furthermore, there is a slightly elusive difference between A_*i *_and B: it seems that A_1 _and A_2 _are *directly *involved in creating new A_1 _and A_2 _entities, while B has only an *indirect *effect on the formation of new Bs. This was captured effectively by Blackmond [[48], p. 386] in the notion of autoinduction: '*autoinduction *[is] where a reaction product or side product accelerates the rate of [...] a reaction sequence without directly producing more of itself. Autoinductive processes may exhibit kinetic signatures similar to autocatalytic processes'. If the reaction sequence is autocatalytic and produces more of this catalytic product in focus, then this product behaves the same way as any component of the autocatalytic cycle. This simply means that any entity, that is not part of a cycle, can only be an indirect autocatalyst piggybacking the host cycle. The only exception is where both A and B are part of their own cycles which are coupled in a hypercycle (pictured in Figure 7c). Of course, the major difference between birds and nests is still the informational criterion, discussed in turn.

### Heredity

In order to clarify the difference between informational and non-informational replicators, heredity must be explained. For this some definitions have to be fixed. *Copying *should be defined as a process that can recreate an entity in such a way that any change applied to the original reappears in the new; thus it should be defined in terms of *heredity*. Heredity should be defined as the potential to pass on changes, in terms of *variability*. Also variability (and, thus, heredity and copying) should be defined only for *modular *entities, since without modules there are no elements of an entity that can change (and be inherited) such that the entity itself is not rendered functionless.

Again, it is instructive to begin with the simplest possibilities of information storage in chemical systems [49]. In this context, molecules act as signs. One can store information with the quantity of signs, with the relative proportion of signs and with the fixed geometric arrangement (for example, sequence) of signs. If the stored information, in unaltered or changed form, can be passed on to the progeny, we then have to deal with the phenomenon of inheritance. It is instructive to look at some unconventional examples in order to illustrate the power of these distinctions.

In the simplest chemoton model of Gánti [49], consisting of three autocatalytic subsystems (metabolism, template, membrane), the simplest form the template macromolecule consists of *n *pieces of monomer V and, thus, it is a homopolymer. The only property that can change is the degree of polymerization (*n*). It was shown that such a mutation (deletion or insertion) affects the functioning of the whole chemical supersystem (via mass action) in a hereditary manner [50]. We are, therefore, dealing with a case where the quantity of a chemical sign carries information. If one passes now to a system, analogous to nucleic acids, where one has polymers with the structure p(VS)_*n*_(WZ)_*m*_, and where capital letters denote different monomer types, then such a template can also carry information with the relative proportion *n*/(*n *+ *m*) of signs. Although, in this case, the sequence is also inherited, it is not yet utilized in the system. Sequence becomes important when, for example, such heteropolymers begin to act as enzymes (such as ribozymes).

Let us now look at the case of 'compositional genomes' and 'compositional inheritance', as proposed by Segré *et al*. in the GARD (graded autocatalytic replication domain) model [51,52]. This immediately connects our discussion to the recent excitement of various abstract and artificial protocells [53]. This field is growing fast. Here we illustrate some of the relevant questions with two protocell models. The GARD model depicts a population of molecular assemblies built of amphiphilic chemical compounds (such as lipids). Assemblies can take the form of micelles or vesicles, but this is not important for the dynamics in question. The building blocks are provided in the medium. Assemblies can grow by incorporating molecules from the medium in a catalyzed (fast) and uncatalyzed (slow) manner. If there are *n *types of different building blocks, then one can define a matrix β, specifying to what extent molecule L_*i *_in the assembly catalyses the incorporation of molecule L_*j *_into the assembly (where *i *and *j *run from 1 to *n*). After reaching a critical size assemblies split into two, so that molecules re-assort into the offspring assemblies at random. Segré *et al*. [51] noted that, despite the fact that one only has compositions rather than geometrical arrangements of building blocks, some compositions can persist through many generations in a given lineage. These quasi-stable assemblies have been called *composomes*. The relevant information is thought to be stored in the respective compositional genomes. Somewhat disturbingly, it was also found that, in a lineage, composomes can revert suddenly to any previous state, thus long-term inheritance looked, at the least, questionable.

Vasas *et al*. [54] have re-analyzed the dynamics of the GARD model from, the viewpoint of population genetics. They have firmly put composomes into the category of attractor-based inheritance [40], where the quasi-stable states coincide with internally strongly interacting subsets, via the β matrix, of building blocks. However, there is a snag. Stochasticity in growth and fission generates variation, so that these alternative subsets tend to flip into other subsets (therefore inheritance is not as deterministic, or exact, as in case of template replication). They were able to derive an Eigen equation [55], in which the different species were assemblies with different initial compositions. The metastability of the composomes translates into some giant mutation rates (which can even be higher than the exact reproduction rates). Hence, the population cannot respond to directional selection and there is no phylogeny. The dynamics is effectively determined by the β matrix. Alternative substates are not stable enough to give a large number of different, stably heritable states. Contrast this with the chemoton containing heteropolymer templates without sequence utilization: there the stability of inheritance is due to covalent bonds and template replication.

Finally, here is the last example from the protocell world. The stochastic corrector model of Szathmáry and Demeter [56] deals with a bag of unlinked genes, the dynamics of which can also be cast in terms of an Eigen equation (where initial gene composition is the relevant species trait). In contrast to the GARD model, a population of such protocells can respond to directional selection, unless there are too many genes in the protocell (when group selection becomes ineffective and, also, advantageous mutations are diluted out). The reason for this difference is that the genes are stably passed on as units (their monomers cannot segregate from each other), whereas the subsets of compositional genomes are not covalently stabilized entities.

The case of membrane inheritance is an interesting, existing, case of alternative forms of hereditary information in biological systems [26,27,57]. Several membranes (such as the cell membrane, the mitochondrial and plastid membranes, and others) are obligate autocatalysts: there needs to be a piece of specific membrane in place for the membrane to grow while maintaining its identity. This is due to the fact that there are specific import machineries in the different membranes that only pull in cognate macromolecules. The key autocatalytic component is that the import machineries also import components of themselves. One could totally confuse the cell by re-distributing these import machineries among the different membranes, while not touching the genes at all [28]. This is a case of limited heredity that, nevertheless, is of high importance for the maintenance of the living world. Of course, the components of the import machineries are informed by genes; but the correct completion of their self-assembly requires an autocatalytic mechanism that is not in the genes. Occasional losses or gains in the number of different hereditary membranes were critical membrane mutations during the history of the cell [27]. This raises the question: what kind of replicator is a hereditary membrane? The answer has been given by one of the authors: 'There is something peculiar about membrane inheritance. Newly synthesized proteins are recruited on the basis of a very limited aspect of their molecular phenotype, namely the presence of the cognate signal peptide, whose primary sequence is usually not conserved. A template-like effect does play a role in this recognition process (the shape of the membrane receptor and that of the signal peptide of the imported molecule must be sufficiently complementary) but heredity is limited. Genetic membranes are ensemble, phenotypic replicators [...]. They are not attractor based because their identity also requires genes, external to them' [[25], p.1674].

Lastly, the case of prions must be discussed in the light of recent findings. Prions are infectious proteins capable of replicating by forcing their alternate conformation (and, therefore, alternate function) on the normal physiological form of the protein (both having the same amino-acid sequence). Originally prions were considered to be simple replicators which were only able to convey one bit of information: normal or infectious conformation. Later, various other strains were identified possibly with different conformations. Recently Li *et al*. [58] performed a series of experiments to demonstrate that different selective regimes (for example, the presence or absence of a prion inhibitor) cause the propagation of different prion strains. Therefore, prions have various phenotypes and these phenotypes cause differential survival, rendering them to be units of selection. In order for prions to be units of evolution, it is necessary that mutations appearing in their conformation are stably heritable. Li and co-workers have found that new variants appeared during replication in the prion population (instead of being there in an initially heterogeneous population), with an assumed (lower limit of) mutation rate of 10^-6^/doubling - although they do not exclude the possibility that new variants are identical in conformation, differing in some other factor (for example, some small cellular RNA determinant associated with the protein). Nevertheless, if any such determinant is passed on to offspring (during infection new determinants are associated with the infected proteins), we are dealing with at least limited heredity. Of course, if the conformation is the same, then only the determinants convey the information; while if there are no determinants, the protein conformation is solely responsible for the evolution of the various prion strains. It would be interesting to know whether the different strains and substrains can mutate into each other (and with what rate) or whether only the native prion can mutate to infectious conformations. It must be noted, of course, that if there is no supply of native prion proteins via gene expression, no invasive conformation can propagate in the population.

What we understood from all the above examples is that heredity must always be a result of copying, and copying is only available in case of modularity. Szathmáry has stated that for the multiplication of holistic replicators: ' [...] replication is not template replication (copying) that rests on a modular polymerization of monomers' [[25], p. 1672]. In case of alternatives of a cycle (see [39]), the intermediates are not copied (being holistic), but the system as a whole may exhibit attractor-based heredity [40].

Only those parts of an entity contribute to heritable information which can inherit changes acquired during the lifetime of the entity. Thus, we call any change heritable that can be passed on to next generation. It is possible that only such changes were gathered during the existence of the replicator that are not passed on during replication (see DNA methylation, isotope substitutes in a replicator, and such-like). The part that can inherit changes is called the *genotype *of the replicator. Note that, in case of epigenetic inheritance, the epigenetic information that is inherited is indeed epigenetic, but not *epigenotypic - *that is, it is part of the abstract *genotype *of a replicator, more than the genome. To be more general: if there is a heritable somatic information, the *genotype *of an organism is more than the information in the germline. The exact definitions, therefore, are:

2.1 *Copying: *a multiplication process of some part of an entity, where there is a potential that any change made in the part of the original entity can reoccur in the part of the new entity (the part is the *genotype*).

2.2 *Heredity*: attribute of an entity which can multiply a part of it such a way that there is a potential that any change made in the part of the original entity can reoccur in the part of the new entity (the part is the *genotype*, and the process of multiplication is *copying*).

2.3 *Modularity*: attribute of a part of an entity, if this part can acquire changes during the lifetime of the entity in such a way that the entity is not rendered non-functional, nor that will dissociate, and that the new entity is potentially phenotypically equivalent to the original one (the process is *copying*).

2.4 *Genotype: *part of an entity (for example, subset of features) which can potentially pass on changes (acquired during lifetime of entity, incorporated into the said part) to offspring during a multiplication process (the process is *copying*).

Note that the definitions refer to equivalence and multiplication defined above (1.2 and 1.4). Modularity in this form fits both heritable and non-heritable changes. Also note that, if there is no heritable variability, there can be no microevolution. Furthermore, the notion of *genotype *implicitly refers to *copying*, *heredity *and *modularity - *for example, the genotype is always modular.

If a variant of a feature is not passed on to offspring during *copying*, then that variable feature is irrelevant from the viewpoint of information-replication, as it is not part of the *genotype*. However, and this is of extreme importance, any change made to any part of a replicator that is passed on to the offspring is part of its *genotype*. That is, a changed base in a DNA sequence can be inherited, so it is part of the *genotype *of the DNA, and *if *the cut tails of mice were to be passed on to offspring then the state of tail would be part of the mouse's (as a replicator's) heritable *genotype *(but, of course, will not be part of its genome). Note that the exact method of *copying *the *genotype *is irrelevant: the only important thing is the heritability of changes made to the *genotype*. In other words, it is neither the medium nor the method that counts during the transfer of information, but the fidelity of the transfer.

So far our best candidate for a replicator is the highly abstract *genotype*; although with this we only pointed to an abstract part of an entity (like the base-sequence of a gene), and not to a real, physically individual entity (the actual DNA molecule). The problem with this approach is that if the replicator equals the abstract *genotype *then the DNA should not be a replicator, since methylation of the backbone or isotope substitutions are not inherited in the same process. That is, changes made to the structure are irrelevant from the viewpoint of the message encoded in the base order (although it may be not irrelevant from the viewpoint of replication). However, our definition would require that any change acquired should also be inherited. Expanding these terms, one should come to the point that, if the *genotype *is a part of an entity, *copying *is a process in which this entity is involved and *heredity *is an attribute of this entity, then the entity itself must be called the replicator. The (informational) replicator has a *genotype *that can potentially be *copied*, thus possessing the attribute of *heredity*. In this sense, the definition should be formulated as follows:

3.1 *Abstract replicator*: the *genotype *of an entity.

3.2 *Realized replicator*: anything that has a *genotype*.

Note that both 3.1 and 3.2 exclude SACIs, although ultimately exact replicators should be gathered under the term 'replicator', as was discussed above. Until that point we will use this working definition, which is in close agreement with Dawkins' [[4], p. 83] definition: 'I define a replicator as anything in the universe of which copies are made'. Although it induces a new problem, that will be discussed in turn.

### Organisms are informational replicators

N order to point out the other problem of the present replicator definitions, let us quote (1) the commonly accepted definition of Hull [[9], p. 408]: the 'replicator [is] an entity that passes on its structure largely intact in successive replications' and (2) a more recent one from Aunger [[2], p. 73], especially the part about similarity: 'The copy must be like its source in relevant aspects'. These definitions are indefinite due to the terms *largely *and *relevant*. Both are relative and it is not clear to what they refer.

As we have not yet pinpointed the exact difference between true replicators and organisms, it seems reasonable to discover the common features of these entities, just as we did with genes and SACIs. Let us do a simple theoretical test. Consider the case of a simple cell (noting that cells are usually *not *considered to be replicators). A cell incontrovertibly passes on *some *of its structure 'largely intact' to the offspring cell during division. Also, parent and offspring are similar in 'relevant aspects'. The obvious objection to this statement would be that a cell cannot pass on every change acquired during its lifetime - like the full cytoplasm and membrane configuration (for sake of clarity we set aside those exceptions where, for example, membranes can inherit structure (see [59]) and the fact that actually some aspects of the cytoplasm are inherited (see epigenetic inheritance systems [60,61]). Thus, there is *some variation *in a cell that *cannot *be passed on to offspring cells.

If we return to one of our favourite replicators, the DNA sequence (in a Spiegelmanian setup [62]), we can state that, despite copying, there are also variations that are not passed on to offspring. For example, the substitution of a carbon atom with an isotope, or a base with a harmless base-analogue, is surely not passed on. Thus, one can see that, in case of genes, there can also be variations that are not being passed on. It is obvious that there is no *real *entity that can pass on its *entire *structure during replication, but there may be theoretical considerations based on such abstract entities. However, based on the definition 3.1 above, one should come to the conclusion that, if everything is a replicator that has a *copiable genotype*, then organisms must also be replicators since, for example, asexual individuals make copies of their genomes for their offspring. Here the information in the genome of the organism is the *copiable genotype*. It is clear that there must be some difference between RNA replicators and organisms or genes and cells, and we aim to discover this difference.

One solution could be that, in the case of cells (and, in general, of organisms), a new offspring is created not by copying but by development. It is still true, however, that both genes and cells have some part that is inherited by copying. In the case of genes it is the base sequence of the gene - in the case of (asexual) cells it is the base sequence of the genome (omitting epigenetically inherited traits). The process that creates the non-copied (non-heritable) part of the new gene, or the new cell, can be anything, it could also be through development. In the case of cells, development from this viewpoint is merely the processing of the information that is passed on. This is analogous to the statement that, in the case of a DNA sequence, it is irrelevant what causes the variation that is not passed on to the next generation (for example, methylation or any kind of change made to the macromolecule). This means that the potential similarity of the hereditary part of multiplying entities does not depend on the actual way that the non-heritable part is generated. There are, of course, alternative explanations of differences between replicators and organisms based on development, but Griesemer's reproducer definition [6] fits our concept quite well. He classifies multiplying systems on a material basis [19], while we focus on the informational aspect. The two classifications may cooperate: an entity may acquire its materials from a different source than the template information on the way to assemble the material.

The seemingly massive discrepancy of the absence or existence of development can be tackled somewhat differently, demonstrating that *developing reproducers *(for example, organisms) are not so very different from *replicators*. Imagine that the quantity of the structure created by development during replication (using the inherited part) can vary from entity to entity. It can span from a considerable amount, as in case of organisms where the whole soma is developed, to nothing, as in case of the idealized gene where hardly anything is developed. Consider a replicating DNA where the offspring simply copies and inherits the base-order, but the recreation of, for example, the sugar-phosphate chain is by some kind of primitive development, using mainly the inherited information (as is the case of RNA molecules, which acquire secondary structure by 'using' the information of the base-sequence). Would not it be considered a replicator? The bottom line is this: as long as there is a *genotype to copy*, the entity incorporating the genotype is a replicator, regardless whether or not development is involved.

Although it is clear that one cannot advance much further with the introduction of development, we can still formulate a few conclusions based on the present position:

**1**. Any entity increasing in number (be it a cell or a gene) has some similarity to its offspring.

**2**. There are parts in both real genes and organisms that can inherit acquired changes and parts that cannot; thus both the cell and the DNA can pass on some of their information content and none can pass on its full information content.

**3**. From the viewpoint of the recreation of the heritable part, the cause of variation in (and the recreation of) the non-heritable part is irrelevant. Note that the recreation process of the heritable part is copying, while the process recreating the non-heritable part *cannot be copying*, otherwise it would be part of the heritable part.

**4**. Both cells and genes fulfil the causality criterion (that is, the parent is involved in the creation of the offspring).

Although it seems appealing to use only the first statement as a criterion for replication, we have to resist this route, as Griesemer [[19], p. S356] has pointed out: 'surely replication means more than just [non-zero parent-offspring] correlation'. If this were the case then even a divided glass of water or a piece of bread cut into half would be a replicator.

With reference to the second statement (and Hull's original definition with the term 'largely'), it is presupposed that the difference between genes-as-replicators and organisms-as-replicators must not be qualitative to begin with, but quantitative. It is this quantitative difference that is the cause of the emergent qualitative differences between gene-like replicators and organism-like replicators. Since a highly complex entity (an organism) cannot be copied entirely to give rise to offspring (see 'organisms are not replicators: they do not reproduce by copying' [[36], p. 556]), some part of it must be developed in order to gain the necessary similarity and to preserve *equivalence*, while some part of it is obviously still *copied *(the genome, or at least parts of it). By development, we refer to a process, which primarily does not imply the copying of information, but is the interpretation of information which had possibly been copied earlier.

### Variability - heredity model

If we accept the expansion that SACIs, organisms or even populations, can be thought of as replicators, a method should be presented that can handle and discriminate among these multiplying entities based on a general model.

**Figure 10:**
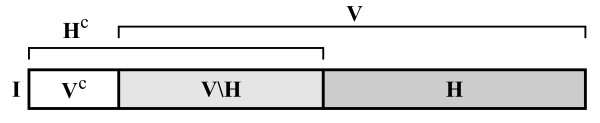
**Ordered informational scheme of a multiplying entity**. I is the complete information set of the entity, V is the subset of I that can change, H is the subset of V that can pass on changes to offspring.

Let the full information content of an entity *E *be denoted by I. Thus, I can be imagined as the complete set of features of an entity -that is, all those properties which are relevant for the observer. Part of the entity is subject to change during the lifetime of the entity (*within-generation *change). It is denoted as V ⊆ I. V can be imagined as the set of those features that can change state, while the functional identity of the entity is maintained. This calls forth a subset of I that cannot change, V^c^. One can think of this as a set of conservative features, which, if changed, renders the whole entity nonfunctional or even causes its decomposition. Furthermore, there is a heritable part of the entity H ⊆ I, that can acquire changes and can pass these changes on to the offspring (the *genotype*). It is pointless to think of heritable parts inside the non-variable part, as non-variable features that cannot pass on variability and that heredity is defined only in case of variability (1.4 and 1.1). Therefore, H ⊆ V. Let *I *be the size of I, while *H *and *V *denote the sizes of sets I and H as fractions of *I*, thus 0 ≤ *H *≤ *V *≤ 1. Figure 10 gives a basic scheme of the subparts (sets of features) of any replicator.

**Figure 11:**
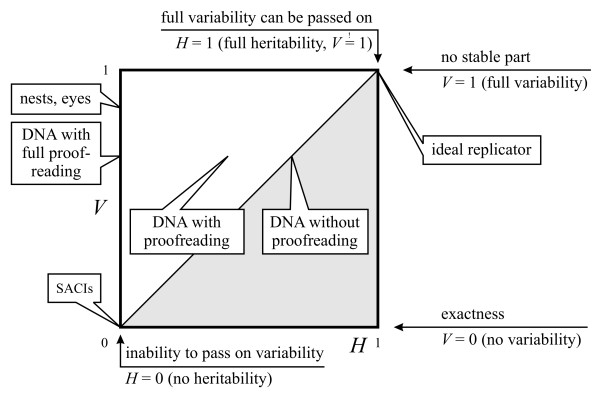
**Semantic domains of the *V H *space**. *V*: variable part size; *H*: heritable part size. By definition: *H *≤ *V*.

If we consider that *I *is unity, *V *can span from 0 to 1, while *H *spans only from 0 to *V*. At *V *= 0 entities do not have variability or heredity at all (*H *is not defined - it could be, for example, SACIs). At 0 <*V *= *H *< 1 are entities with some variability and full heredity. They are capable of passing on every acquired change (since *V *- *H *= 0). As a special case, an entity at *V *= *H *= 1 can have changes anywhere in its structure (without becoming functionless); thus it is a compact entity and can pass on every change (this would be a hypothetical *ideal replicator*). Note that *V *<*H *is not possible due to the definition of heredity. Figure 11 shows the distinction of replicators according to *V *and *H *(a slightly similar classification was derived by Solé [[63], p. 282], where spatial and informational complexities of replicating systems can be paralleled with *V *and *H*, respectively).

The model suggests that, if we accept that entities described by the *V H *model are all replicators, we should include SACIs, nests and organisms in the group of replicators. On the other hand, it still allows the exclusion of such entities, given that they are extremes of the complete space. The difference is quantitative, but now we have the factors needed to measure the difference of this variability and heritability.

Examining the model, a statement can be expressed as: *exactness *is not the same as the *inability to pass on variability*, although both show exact replication. *Exactness *is an attribute of a replicator, which cannot have new (acquired) variability to pass on and does not have errors when copying (in general it is variation-free). The inability to pass on variability is an attribute of a replicator that can have variability (acquired or generated), but it cannot pass on any of its variability to the next generation. Neither has a definite genotype. So the two attributes are built of three factors, from which the latter two are only available if the first ability is present:

**1**. The ability to acquire variability during lifetime (*V *> 0).

**2**. The ability to pass on acquired variability during multiplication (part, *H *> 0).

**3**. The ability to generate variability during multiplication (erroneous copying, *μ *> 0).

The classification of these replicators is summarized in Table 1.

The *V H *model can predict the overall structure-similarity between parent and offspring - that is, it can predict the quantitative difference between *E*_*p *_and *E*_*o *_(of parent and offspring, respectively). For this we have to introduce two more variables: *υ *is the probability that a feature (or a module) of the variable part changes during a unit of time, and *μ *is the mutation rate suffered during the replicative process (the probability that a heritable feature is miscopied). Parameter *υ *is the usual number of effective changes acquired in the time period between two replicative processes. The overall similarity *S *(the total number of identical features) is a distance function *f *of parent and offspring entities (taking into account only the time spent since the last replication of the parent):(4)

**Figure 12:**
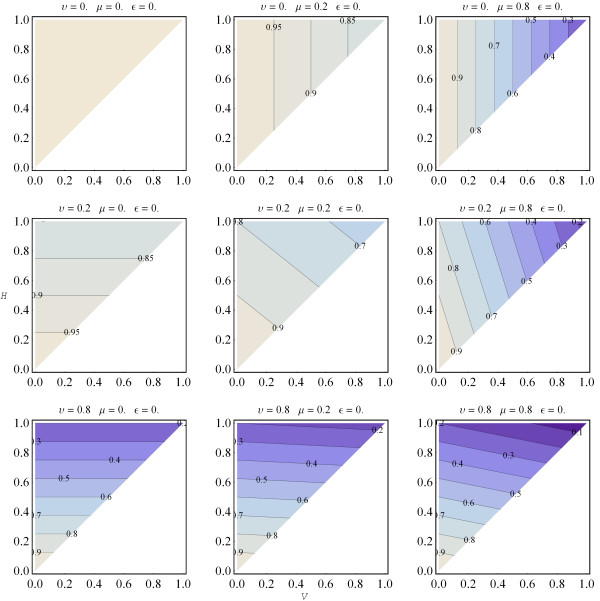
**Contour plots representing similarity values between parent and offspring entities (both being modular) as a function of *V *and *H***. **Parameters *υ*, and *μ *are mutation rate during lifetime and mutation rate during replication, respectively**. The chance of backmutation is taken to be negligible (*ε *= 0). By definition *H *≤ *V*.

*I*_*p*_, *V*_*p *_and *H*_*p *_refer to the appropriate parts of *E*_*p*_. The first term (*I*_*p *_(1 - *V*_*p*_)) is the contribution of the stable part of the entity to the overall similarity. The second term (*I*_*p *_(*V*_*p *_- *H*_*p*_) (1 - *υ*)) specifies the non-changed variable but non-heritable part of the offspring. The third one (*I*_*p *_*H*_*p *_(1 - *υ*) (1 - *μ*)) is the contribution of those modules, that are members of the heritable part and are neither changed during lifetime nor miscopied during replication. The last term (*I*_*p *_*H*_*p *_*υ μ ε*) specifies those heritable modules that are changed during the life of the parent and miscopied in such a way that they are backmutated to the original state. The parameter *ε *gives the probability of this backmutation per module per replication. This depends on the inventory size of the medium the genotype uses. If the inventory size is large, then the probability of a changed module mutating back is fairly small, even negligible (*ε *= 0). If the inventory is small there is a considerable chance (or complete certainty in the case of a binary inventory) that the result of the mutation will be the original one (since mutating an element twice will result the original one). It is obvious that in the case of *υ *= 0 or *μ *= 0 the entity can only replicate exactly (that is, it is potentially a replicator, but actually is lacking variability, perhaps due to the environment). Thus, if *I *is taken as 1, similarity depends on five factors: 0 ≤ {*V*, *H*, *υ*, *μ*, *ε*} ≤ 1 and, by definition, *H *≤ *V*. Figure 12 shows the change of the similarity surface in case of various (*υ*, *μ*) parameter combinations.

The problem, in the case of organisms, is whether they exhibit an overall similarity high enough to count as replicators. This is a quantitative question, as it was pointed out earlier. Preliminarily, we suggest that the distinction between simple replicators (such as genes) and organisms be based mainly on two factors: first, the presence of development; and, secondly, the separation of two parts - one that can be changed and inherited and another that cannot be inherited and is highly stable. Rephrasing this second criterion we see that *ideal replicators *do not have non-heritable but variable parts. Furthermore, a replicator should minimize the fraction of its non-heritable but variable part (that is: (*V *- *H*) → 0) if it wants to approximate ideal replication. Therefore, the abstract, ideal replicator should not have any non-heritable variability in itself - and this is suitable only for the abstract *genotype*, defined in 2.4. The more we loosen this restriction the closer we get to a real gene (that has a very low variability in its stable part, the sugar-phosphate chain) and increasing the (*V *- *H*) fraction eventually brings us to (for example, asexual) organisms, where the non-copiable but variable part (the whole soma) is a much larger part of the whole entity than the genome itself (that is a part of the germline).

### Classification

**Figure 13:**
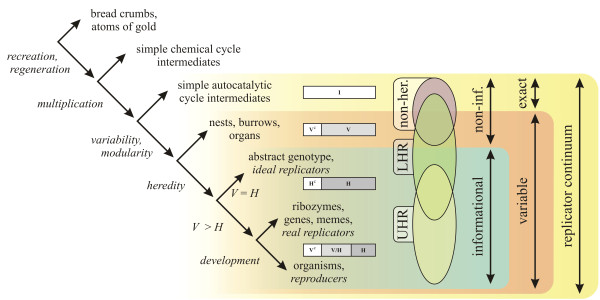
**Hierarchy and classification of multiplying entities**. Each downward arrow introduces a new feature. Inset diagrams show a vague concept of the parts of the given entity regarding the *V H *model. Obviously, the replicator continuum can be categorized based on various aspects, for example variable/exact, modular/non-modular or informational/non-informational, unlimited-/limited-/non-hereditary, and so on.

As demonstrated, the diverse set of multiplying entities may include various entities, which, somewhat arbitrarily, can be termed replicators. To overcome this arbitrariness, a hierarchical classification is presented focusing on the relation of these entities (Figure 13).

A regenerating/recreating entity can produce at least one entity equivalent to it. It is possible that the original entity immediately decomposes (that is, cannot be the subject of further turns of the cycle), causing sequential replacement, although this is the most simple of regenerating entities. If it can effectively increase the number of entities equivalent to itself, then it is autocatalytic and is a *replicator*. The simplest of replicators is the *exact replicator*, which is *non-informational*, and any change made to it causes a change in the phenotype. If a variation can arise in the structure in such a way that it does not change equivalence of the entity, then it is a *variable replicator*, with more than one stable state. If such changes can be passed on to the offspring then the replicator is *informational*. If the non-heritable part is constructed by a developmental process, then the replicator is a *reproducer*.

The classification implements the fact that each subset acquires some new attribute for which all the previous attributes are needed. For example an (asexual) organism is therefore a *reproducer*, a complex replicator, which has a genotype, and the non-heritable part is built up by development. Note that the evolved process of development is able to increase the similarity of the variable but non-heritable part of an entity compared to its parent entity, which helps (and is assumed to evolve) to maintain the equivalence of successive entities.

The housing of genotypes by more complex entities also implies an obvious fact. The actual, real, physically individual replicator is the most compact physical part of an entity that approximates the abstract genotype best. This means that there can be hierarchical containers (vehicles [64]) encapsulating the abstract replicator within different levels.

An asexual organism is, thus, a replicator by this definition. It is more convenient, however, to restrict the term only to that most bare-boned, individual part of the organism, that still holds the abstract genotype (that is, to minimize the variable but non-heritable part) - the genome or the set of chromosomes. Even more convenient would be to refer to the *abstract genome *(the base sequence) as the replicator, since the abstraction of the genome is the genotype. Nevertheless, it is merely a matter of viewpoint whether one thinks about organisms, genomes or base sequences as replicators, until the referred genotype is the same. In sexual cases, it is clear that the genome cannot be the genotype as a whole, since genome *g*_1 _of one parent will be halved and complemented from another parental genome *g*_2_, to form the offspring-genome. Thus, the presupposed genotype (*g*_1_) is actually not the genotype, because, in this situation only, the actually inherited set of genes forms the genotype. This is why Dawkins has introduced the gene's eye view concept [3] contra the genome.

### Evolution

An important question follows: which changes (heritable or not) affect the outcome of selection? Let us return to the term of 'relevant aspects'. Could it be that it is only the genotype that is relevant? If we suppose that the phenotype is the identity function of the genotype (in the mathematical sense: *p*(*e*) = *g*(*e*)) then the situation is simple: similarity is only affected by those changes, that alter the genotype (and not other aspects of the replicator) because these directly affect the phenotype as well.

The amount of hits the genotypic equivalence can stand was given formally by Eigen as the error threshold of the quasispecies model [55]. It clearly gives a lower limit of the quantity of changes with which the original sequence can still be maintained in a population, where it is surrounded by its Hamming-neighbours. The problem with this approach is that there can be changes in the replicator that do not affect the genotype but do affect the replication/survival rates. Thus, a change in the phenotype does not necessarily go along with a change in the genotype.

In Eigen's [55] framework, the simplicity of the model comes from the assumption that it is only the genotype that directly affects the replication rate (remember, this was our presupposed condition at the start of this section). It is more or less true in case of simple biosequences in a Spiegelmanian experiment [62]. It is usually only the base-sequence (the genotype of the replicator) that is changed, and, thus, every sequence has the same non-heritable structure (the sugar-phosphate strands).

However, one should not forget that the phenotype of an entity is not necessarily an aspect of the same physical structure that encodes the genotype (as in case of Eigen's framework). A gene may have its phenotype at the level of proteins, or at the level of the organism in which it is housed (the vehicle), or could even have phenotypic effects on other organisms concerning the actual level of selection (see extended phenotype concept of Dawkins [[64], p. 55]), although it must be emphasized that phenotypes (and therefore fitness) are specific for a certain level of selection, for a certain environment. Following on from the supposition that genotypes and phenotypes may have different media (that is, the genotype of a gene is manifested in nucleotides, while the phenotype is in proteins/behavior/and so on), it can be stated that there could be, and actually are, such non-genotypic changes that affect the replication rate (the phenotype). Table 2 summarizes the implications of various changes.

**Table 2:** Effects of acquired changes on a replicator

		*Change phenotype?*		
		**No**		**Yes**
	**No **(for non-informational replicators)	**1**. Non-heritable neutral change		**2**. Non-heritable effective change
*Change genotype?*				
	**Yes **(For informational replicators)	**3**. Heritable neutral change		**4**. Heritable effective change
		Trivial neutrality Nontrivial neutrality		

We have already seen that for non-informational replicators only those changes affect the outcome of selection that changes the phenotype [for example, the kinetics (2) in Table 2]. Although, since these changes are not heritable, they have a one-time effect, which may imply some influence on long-term selective situations. In informational replicators it is possible to have genotypic changes that do not change either the phenotype [(3) in Table 2], and phenotypic changes that are results of changes in the genotype [(4) n Table 2]. The latter is the main drive of evolution, while the former is almost equally important. Heritable neutral changes are the primary causes behind sudden evolutionary changes. The genome collects neutral mutations which (at one point) overload the robustness of the system, ultimately inducing a shift in the phenotype space (see [65]). Non-heritable neutral changes [(1) n Table 2] do not have any effect either on selection or on evolution.

Note, heritable changes that do not have an immediate effect on the phenotype may affect the possible phenotypic distribution of mutants of the genotype (non-trivial neutrality), and therefore affect the phenotypic exploration space and ultimately change the long-time evolutionary potential [33,66].

It must be emphasized that until changes do not affect the phenotype of the replicator, selection acting on the phenotypes will not induce the indirect selection of genotypes. This means that, if there is no selection, neither genotypes nor phenotypes are evolvable (actually, the phenotype is not even defined) since all replicators will be equivalent. In contrast, entities may accumulate changes in their genotypes that may be called neutral microevolution. Again, the crucial point is that without anything linking a changing genotype to a change of the phenotype, no real evolution in the phenotype-space can be present. This is consonant with the fact, that direct evolutionary changes are those that change the reproductive and/or survival success of the given replicator (see, for example [34]).

In turn, if there is no link between phenotypes and genotypes, then selection on the level of phenotypes cannot change the distribution of genotypes - that is, it cannot cause indirect selection of the genotypes. This is more emphasized in situations where the phenotype is directly manifested in a different entity than the genotype, for example the gene-protein system, where (for sake of simplicity) the protein can be thought of as the entity that is under direct selection. Ruiz-Mirazo *et al*. ([67]) grasped it as a partial decoupling between the genotypic and the phenotypic domains, which in turn allows for the development of free compositionality and the gaining of a higher complexity at the level of genotype - that is to open wide the door for open-ended evolution.

For a more formal description: let *E *be an entity which can be described by the information vector *e*: {*e*_1_, *e*_2_, ..., *e*_*n*_}. Then let *G*_*e *_be a subset of *e*, the elements of which are responsible for the genotype *g *such that: *g*(*e*) := *h*(*G*_*e*_). Similarly *p*, the phenotype, is defined as: *p*(*e*) := *f*(*P*_*e*_), where *P*_*e *_⊂ *e*. If it is true that *G*_*e *_and *P*_*e *_do not have any common element (that is: ∀ *x *∈ *G*_*e*_: *x *!∈ *P*_*e*_), then no change in the genotype can induce a change in the phenotype, while selection on the level of phenotypes cannot be channelled toward the genotypes. Thus, any change affecting the genotype would be neutral from the viewpoint of selection.

Therefore, the replicator in the evolutionary sense must have some additional attribute. There must be a connection between genotypes and phenotypes, such that the genotype should channel acquired changes toward the phenotype which, in turn, should cause an indirect selection among genotypes. There can be a variety of mapping functions from genotype to phenotype. A completely bijective mapping would cause a one-to-one mapping from a different genotype to a different phenotype, while the standard genetic code maps 64 codons to 20 amino acids and the stop signal. Of course, the nature of the mapping, while not effecting selection directly (as neutral genotypic changes will not affect the phenotype), has major implications for the evolutionary potential.

The dyadic approach of changes (genotypic/phenotypic) implicitly suggests that, in the similarity of informational replicators, there is a second aspect which may be relevant apart from the phenotype (and selection): the genotype (and evolution). From the viewpoint of selection, any change affecting the replication or survival rate (the phenotype) is relevant. From the viewpoint of evolution only those changes are important which alter the genotype but, since adaptive evolution cannot proceed without selection, these changes also have to alter the phenotype (otherwise they are neutral mutations). Note: selection primarily does not involve evolution in the sense that entities accumulate changes through time.

This allows one to expand the definition of replicators to a broader view where replicators may be subject to not just selection but evolution as well. This was, of course, already pointed out by the distinction of non-informational and informational replicators. The former cannot undergo microevolution, although a diverse population of them can be a subject to selection. The latter, which possess heritability, may open the door for unlimited evolution.

It seems logical here then to formulate the definition of entities that can be units of selection and units of evolution:

5.1 *Unit of selection*: an entity is a unit of selection if there potentially exists a population of such entities, in a particular environment and, according to a particular selective force (for example, resource limitation), they are not equivalent, possessing different phenotypes.

5.2 *Unit of evolution*: a replicator is a unit of evolution if it is a unit of selection and has a heritable genotype that is linked to the phenotype.

The unit of evolution incorporates all the criteria of multiplication, heredity and variability implicitly. It must be noted that only an informational replicator can be a unit of evolution, since, for evolution, a genotype is needed that can copied time to time. Note: the unit of selection is not exactly the one introduced by Maynard Smith [30]. Here, a unit of selection does not need multiplication, although multiplication and variability were the Mullerian criteria used by Maynard Smith. It can be argued that multiplication is inevitable for units of selection. Since selection was defined simply as sorting that causes differential survival of entities (definition 1.1 above), one can say that selection can sort among a population of entities without any kind of reproduction. A consumer may select among goods by their price or quality (phenotypes), if the chosen factor exhibits some variation. However, without the multiplication of these entities, selection would be a one-time only event. The unit of selection in the sense of Maynard Smith (multiplication and variability, but no heredity) obviously denotes non-hereditary replicators.

The notion of the evolutionary unit (or informational replicator) is still not enough to distinguish larger chunks of the genome from short oligonucleotides. It is unlimited hereditary that is indispensable for open-ended evolution [17], as discussed earlier. Larger biosequences possess this, while shorter ones (and attractor-based systems) can only have a limited hereditary potential.

## Conclusions

Criteria of the replicator were discussed and the nature of its functions (genotype, phenotype), attributes (modularity, variability, heritability, exactness) and processes (multiplication, copying, heredity, and selection) were exactly defined. According to the implications of these factors, the following definition is proposed:

*Replicator*: any autocatalytic entity for which there is a selection process defined. Selection is a process, acting on a particular population of entities in a particular environment, which sorts entities according to their phenotypes.

This includes informational as well as non-informational replicators. In Mullerian terms, it simply requires multiplication and variability (that is, there is a difference in the phenotypes of a population of such replicators) criteria, but not necessarily heredity. For informational replicators (replicators with explicit genotypes) the definition can be further detailed a bit:

*Abstract informational replicator *(3.1): a replicator in the narrow sense: the abstract genotype itself that is multiplied. The genotype is that part of an entity which can potentially pass on changes (acquired during lifetime of entity, incorporated into the said part) to offspring during a multiplication process, called copying.

*Realistic informational replicator *(3.2): a replicator in the broad sense: any multiplying entity that has a heritable genotype - that is, the smallest entity that houses the genotype.

Using these definitions the first question - What is the difference between entities such as genes, organisms, SACIs and other reproducing entities? - can be answered clearly. SACIs are replicators, although non-informational, because they multiply autocatalytically, but lack a genotype. They cannot accumulate changes during a lifetime that can be passed on to offspring. With this restriction one can also classify those entities under non-informational replicators, which behave like SACIs but are more complex (such as nests, burrows and organs - but only if they cannot propagate information to offspring, unlike in the vegetative reproduction of plants). This means that the inheritance of information equals the possibility of inheriting changes acquired by the parent during its lifetime. Furthermore exact and variable replicators were identified (without or with the possibility of variability) in order to classify SACIs and organs/nests, respectively. Whether we think of these as part of the complete replicator-continuum or not is a matter of arbitrary decision. Nevertheless, the hierarchy is absolute.

A related question is - What are the unit of evolution, and the level of selection? Dawkins' statement about the gene being the sole unit of evolution (gene's eye view [4]) is in coherence with our approach. From all possible units (species, populations, individuals, genomes, genes, and so on) the gene is the one that best approximates the *abstract informational replicator*, minimizing the variable but non-heritable part.

Also it was demonstrated by formal stoichiometry that those heterocatalysts of an autocatalytic cycle which are multiplied by the cycle are actually indirect replicators with autoinductive behaviour [48]. Therefore, they are facultative autocatalysts, although it must be emphasized that they can only be dependent replicators, since they cannot be the sole initiators of the autocatalytic cycle. They must meet other catalysts (possibly real autocatalyst) as well, to form a sufficient autocatalytic seed. Consequently, nests (also burrows, organs, and others) are dependent non-informational replicators, which may put an end to an ancient debate about whether a 'bird is the nest's way of making another nest' [42], suggesting that the nest is the replicator. Based on our argument, a nest is, indeed, an autocatalytic replicator (although a dependent one), but it is also true that, just as there is no causal arrow from bird to gene [[4], p. 98], there is no causal arrow from nest to bird or from nest to gene, since nests cannot feed information back to the bird, being primarily non-informational.

Our final framework, therefore, differs significantly from that of Dawkins. By applying the useful tool of 'formal chemistry', the replicator concept was extended. We state that not just the non-informational replicators, but also organisms of higher complexity, count as replicators; we also argue that heterocatalytic products of autocatalytic cycles are also replicators (nests).

The bottom line of this argument is that replication, in general, is basically autocatalysis (see Orgel [23]), with a specific environment and selective force defined. Although, autocatalysis does not equal informational replication, the latter is a special case of the former. Informational replication is a certain kind of autocatalysis, where acquired changes are inherited in form of the genotype (see [11]). If no information (that is, change) can be inherited, there is no genotype and no template-based heredity. For clarification, the specifics of informational replication, copying, heredity and modularity were defined exactly (thus answering the second question: What are copying, heredity, and similarity, and what part do they play in case of replicators?).

When do we say that two replicators are identical? The whole notion of parent and offspring entities bearing any resemblance (that is, being similar in some relevant aspects) is interesting only if there is something explicitly differentiating among replicators - if there is any selection going on. Otherwise competition and extinction, or even identity, cannot be defined as a replicator. Selection was thus defined as a sorting function or, more precisely, an equivalence relation applied to a set of entities. Selection, of course, can be any arbitrary sorting function not just natural selection. The function of entities, where selection partitions the set of entities, is the phenotype. Equivalence was defined as the equality of phenotypes. Thus, equivalence is specific for a certain selection in a certain environment. This means that two replicators are identical if they have the same phenotypes - for example, the same fitness in the case of a single sorting event. Of course, in successive iterations of selection, these equivalent genotypes may induce different evolutionary pathways. The notion of equivalency has a very important effect: two entities may be completely different, but still equivalent, until they code for the same phenotype. For example, the neuronal representations of a grammatical rule in two language users may differ heavily but they are still equivalent until they code for the same rule, as it is the rule that is under direct selection. This is the reason why it is so hard to pinpoint a meme in the brain. One should not look for identical structures but, instead, for equivalent ones which may differ but code for the same thing.

The phenotype was, in turn, defined as a function of the entity (in a given environment) that 'can be seen' directly by selection. As the phenotype is not a direct function of the genotype, phenotype and selection were also defined for processive replicators, not just for evolutionary ones. Also, for the same reason, it is possible that the phenotype is entirely independent of the genotype. Such replicators are inert to evolution.

Assigning equivalence to a function of the replicator also means that the specification of the replicator also has to include the function (and the level of selection). A replicator cannot be characterized by a simple biosequence or a molecule, unless the level of selection and the phenotype involved are specified. Otherwise one could not decide if two entities are equivalent. Furthermore, as the level of selection is specific for a certain environment, this environment should also be specified. Thus, an informational replicator should refer to the complex entity that includes the genotype, the phenotype, their relationship, the level of selection and the environment for which selection was defined.

With reference to the question 'What are the common and differing aspects of genes and memes?*'*, not much has yet been said: this matter will be detailed in more depth in a future paper. Nevertheless, since patterns of information are propagated between brains in a way that is analogous to template copying (that is, imitation), we argue that memes are indeed informational replicators. We are aware of the fact that cultural learning is not always based on direct imitation but, rather, on inference and reconstruction (see [14,20]), but we argue that even reconstruction can be thought of as a way of pattern-copying during which information is explicitly replicated.

A model was presented in order to distinguish among entities which are able to recreate themselves and do or do not possess variability and heritability. The conclusion of the model is that the distinction between organisms and genes as replicators, in terms of copiable variability, is mainly a quantitative one, depending on the ratio of the heritable part and the non-heritable (but variable) part. This distinction depends entirely on the actual parameters of the entity (the ratio of its variable and heritable parts, *V *and *H*, respectively) and on the actual parameters of the environment (the probability to change between two replication processes: *υ*; the probability to miscopy a feature during replication and *μ*; the probability of backmutation to the previous state, *ε*). As it was stated, a multiplying entity approximates the ideal replicator (the abstract genotype) by decreasing the non-heritable variability that it can acquire ((*V *- *H*) → 0). However, if this approach is not viable, the replicator can 'try to use' development in order to increase the similarity of the non-heritable parts of parent and offspring. Again, it must be emphasized that the presence of development does not change the fact that a genotype of the organism exists (the abstract information of the genome in case of asexual organisms). The genome of an organism is the analogue of the genotype of a gene (the abstract base-order).

An important recognition was that exactness is not the same as the inability to pass on variability. Seemingly, the two attributes are equivalent, as no entity with any of the two abilities can be a unit of evolution. The inability to pass on variability, however, means that (neutral) variation can emerge in the structure. Thus, it requires modularity in order to acquire changes. Those entities unable to pass on variability simply just cannot make these changes heritable. In contrast, an exact replicator cannot have any variability at all, since any change will change its phenotype.

It has been pointed out that, although material input is needed to build new entities by autocatalytic cycles, no material overlap is necessary. This suggests that every replicator should have some material form, but the information transmission from parent to offspring is not linked to any material overlap and can use any kind of medium (molecules in case of genes, air in case of words, or electronic currents in case of computer viruses).

The viewpoint detailed in this paper (that anything is an informational replicator that has a copiable genotype) brings us to an important new statement. It is not the replicator that is a subgroup of the set of reproducing organisms (such as Griesemer's statement [19], where *replication *was a special kind of *reproduction*), but the reproduction of organisms is a more specific version of replication with the novel ability of development. This hierarchy fits the actual evolutionary history of replication and development even better. Development is a complex process that also involves material overlap and is potentially able to increase the similarity of the non-heritable part of the entity - the part that cannot be copied. The purpose of development from this viewpoint is to increase the similarity to the point where it is possible to further maintain the functional identity (equivalence) of the entity. Griesemer approaches the problem of multiplication from a material point of view [[19], S359]: 'In politics, you follow the money, in biology you follow the stuff'. We took another route. Since Griesemer's classification and ours distinguish between the different aspects, they can stably cooperate (Table 3). This has an important implication: during replication the material and informational parents need not be the same.

**Table 3:** Comparison of Griesemer's classification (material aspect, columns) with ours (informational aspect, rows)

					***Material aspect***		
					**No overlap**	**Overlap**	
						No development*	Development*
***Informational/dynamical aspect***	**No multiplication**			***No replication***	***Transcription, translation***	**Dissipation, segregation (*sensu *Griesemer) *metabolic networks***	***Non-replicating somatic cells***
	
	Multiplication	No heredity		*Non-informational replication*	?	*Formose reaction*	?
		
		Heredity	No development*	*Informational replication*	**Copying **(*sensu *Griesemer) *crystal growth, ribozymes, prions*	*Semi-conservative DNA replication*	?
			
			Development*	*Reproduction*	(*Self-bootstrapping complex entities*)	?	**Reproduction **(*sensu *Griesemer) *cells, organisms, chemoton*

*Our definition of development differs from Griesemer's. It does not necessarily include material overlap, but is taken to be a process during which the final entity is made from the information inherited from the parent (template). Therefore, it is also possible to have entities in the 'development/no development' cells.

Another non-obvious consequence of this definition (both the definitions of the replicator and the genotype) is that it clearly states that acquired changes of the genotype are inherited. If we now expand our focus to include organisms in the group of replicators (as explained above) it should be noted that during the reproduction of such organisms several acquired changes are also passed on (that is, mutations of the genome in the germline and other epigenetic traits). The inheritance of acquired changes was usually associated with Lamarckian, rather than the orthodox Weismannian inheritance. Thus, the redefinition of Lamarckian inheritance is in order. This is a rather complex problem not discussed here, but left for a future work.

It is a clear consequence of the genotypic approach of replication that, unless certain conditions are met, the replicator is not necessarily a unit of evolution. In order to endow the replicator with the ability to become a subject of open-ended evolution by natural selection, it is necessary to link the change of phenotype to the change of the genotype, as the latter is the part which is conserved more or less intact through the generations, while the former is the one that 'controls' the survival of the entity in-between generations. From a mathematical sense it means that the phenotype should be a function of the genotype. Also unlimited hereditary is necessary for a replicator to be the subject of open-ended evolution [17].

The new approach has been used to hierarchically classify multiplying entities, either n order to distinguish them or to collect under one term those that are somehow similar in some features. The model and the hierarchy allow us to classify and diagnose not just present replicators but also future candidates of replicators. It helps to decide whether they really are replicators or whether they are missing some basic criteria in order to be considered as true replicators. The definition will point out the missing requirements and can even be used to predict unknown, but viable, modes of replication.

## Abbreviations

GARD: graded autocatalysis replication domain; N-bird: nested bird; PGA: 3-phosphoglycerate; SACI: simple autocatalytic cycle intermediate; SNCI: simple non-autocatalytic cycle intermediate.

## Authors' contributions

IZ and ES conceived and designed the models and the study. IZ analyzed the models. IZ and ES wrote the paper. All authors read and approved the final manuscript.
